# Aqua­bis­(benzoato-κ*O*)(1,10-phenanthroline-κ^2^ *N*,*N*′)zinc(II)

**DOI:** 10.1107/S1600536810049639

**Published:** 2010-12-04

**Authors:** Ji-Zhong Liu, Zhong Zhang, Zhan-Wang Shi, Peng Gao

**Affiliations:** a Department of Chemistry, Guangxi University for Nationalities, Nanning 530006, People’s Republic of China

## Abstract

The Zn atom in the title compound, [Zn(C_7_H_5_O_2_)_2_(C_12_H_8_N_2_)(H_2_O)], is five-coordinate in a distorted trigonal–bipyramidal coordination environment involving two O atoms of two monodentate benzoates, two N atoms of a 1,10-phenanthroline mol­ecule and one O atom of a water mol­ecule. The axial positions are occupied by a carboxyl­ate O atom from the benzoate ligand and an N atom from the 1,10-phenanthroline ligand [N—Zn—O = 146.90 (7)°]. The water mol­ecule forms an intra­molecular O—H⋯O hydrogen bond; an inter­molecular O—H⋯O hydrogen bond gives rise to a dimer.

## Related literature

For a related structure, see: Necefoglu *et al.* (2001 ▶).
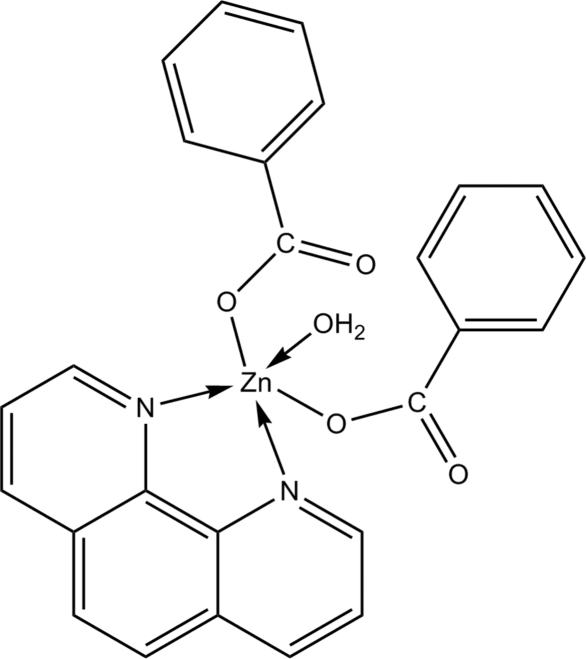


## Experimental

### 

#### Crystal data

[Zn(C_7_H_5_O_2_)_2_(C_12_H_8_N_2_)(H_2_O)]
*M* *_r_* = 505.81
Monoclinic, 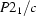

*a* = 10.635 (5) Å
*b* = 21.073 (10) Å
*c* = 11.197 (5) Å
β = 116.647 (5)°
*V* = 2243.0 (18) Å^3^
*Z* = 4
Mo *K*α radiation
μ = 1.14 mm^−1^
*T* = 293 K
0.22 × 0.20 × 0.18 mm

#### Data collection

Bruker SMART diffractometer
Absorption correction: multi-scan (*SADABS*; Sheldrick, 1996 ▶) *T*_min_ = 0.779, *T*_max_ = 0.815
12067 measured reflections
3992 independent reflections
3424 reflections with *I* > 2σ(*I*)
*R*_int_ = 0.026

#### Refinement

*R*[*F* ^2^ > 2σ(*F* ^2^)] = 0.028
*wR*(*F* ^2^) = 0.077
*S* = 1.03
3992 reflections
309 parameters
180 restraints
H-atom parameters constrained
Δρ_max_ = 0.27 e Å^−3^
Δρ_min_ = −0.36 e Å^−3^

### 

Data collection: *SMART* (Bruker, 1999 ▶); cell refinement: *SAINT* (Bruker, 1999 ▶); data reduction: *SAINT*; program(s) used to solve structure: *SHELXS97* (Sheldrick, 2008 ▶); program(s) used to refine structure: *SHELXL97* (Sheldrick, 2008 ▶); molecular graphics: *SHELXTL* (Sheldrick, 2008 ▶); software used to prepare material for publication: *SHELXTL*.

## Supplementary Material

Crystal structure: contains datablocks I, global. DOI: 10.1107/S1600536810049639/ng5072sup1.cif

Structure factors: contains datablocks I. DOI: 10.1107/S1600536810049639/ng5072Isup2.hkl

Additional supplementary materials:  crystallographic information; 3D view; checkCIF report

## Figures and Tables

**Table 1:** Hydrogen-bond geometry (Å, °)

*D*—H⋯*A*	*D*—H	H⋯*A*	*D*⋯*A*	*D*—H⋯*A*
O5—H5*A*⋯O2	0.89	1.78	2.616 (2)	154
O5—H5*B*⋯O4^i^	0.89	1.91	2.797 (2)	174

Symmetry code: (i) 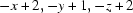.

**Fig. 1.**
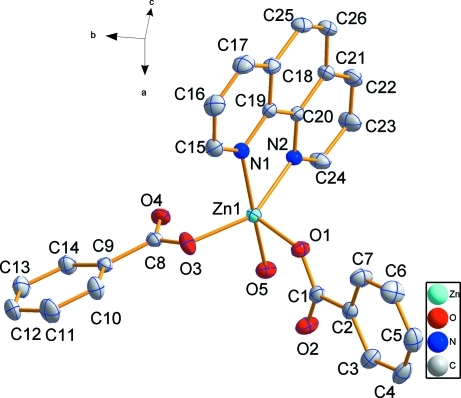
The structure of the title compound(I) and the atom-numbering scheme. H atoms have been omitted for clarity.

**Fig. 2.**
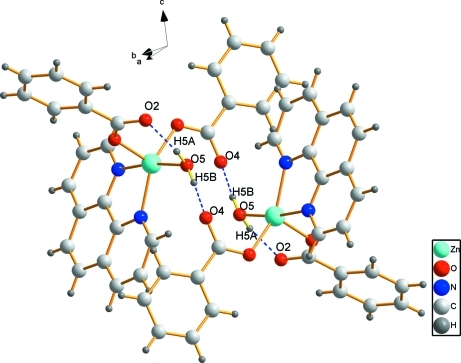
Part of the packing of the title compand, viewed down the direction. Dashing lines indicate hydrongen bonds.

## supplementary crystallographic information

### Comment

In the present study, we report the synthesis and crystal structure of the title complex (I). The biological activity of (I) against common bacterial strains is to be investigated. Selected geometric parameters are listed in Table 1. As shown in Fig. 1, (I) is a mononuclear neutral zinc(II) complex in which the carboxylate group exhibits a monodentate coordination mode. By contrast, in the mononuclear zinc complex of diaquabis (benzoato)zinc(II), each carboxylate ligand forms a primary and a secondary Zn—O bond (Necefoglu *et al.*, 2001). The Zn ion in (I) is coordinated by two O atoms from two monodentate benzoate ligands (O1 and O3), two N atoms from the 1,10-phenanthroline ligand (N1 and N2) and one O atom from the water molecule (O5), and exhibits distorted trigonal-bipyramidal coordination. The trigonal base plane is defined by atoms N1, O1 and O5, and atoms O3 and N2 occupy the axial positions [O3—Zn—N2 =146.91 (5)°]. A strong intermolecular hydrogen bond exists, involving uncoordinated atom O4 of the carboxylate group as an acceptor and atom O5 as a donor (Table 2), resulting in the formation of a dimer (Fig. 2). There is also an intramolecular hydrogen bond between the other H atom of the water molecule and uncoordinated atom O2 of the other carboxylate group (Table 2).

### Experimental

C_4_H_6_ZnO_4_.2H_2_O(0.2195 g,1 mmol), benzoic acid(0.2442 g,2 mmol), NaOH(0.08 g;2 mmol),1,10-phenanthroline (0.1802 g,1 mmol) were added to a mixture of water (15 ml) and ethanol (10 ml). The resulting mixture was stirred at 70 for 4 h and fltered off. The filtrate was allowed to stand at room temperature and slow evaporation afforded colorless block crystals of the complex(Yield 65%).Elemental analysis: found C,61.69;H,3.98;N,5.57;calc for C_26_H_20_N_2_O_5_Zn:*C*,61.73; H,3.99;N,5.54(%).

### Refinement

H atoms on C atoms were positioned geometrically refined using a riding model with C—H=0.93–0.96Åand*U*_iso_(H) = 1.2*U*_eq_(C),The water H atoms were located in difference density Fourier maps and refined using a riding model with O—H=0.82Åand *U*_iso_(H) = 1.5*U*_eq_(C).

### Crystal data

**Table:**

[Zn(C_7_H_5_O_2_)_2_(C_12_H_8_N_2_)(H_2_O)]	*F*(000) = 1040
*M**_r_* = 505.81	*D*_x_ = 1.498 Mg m^−^^3^
Monoclinic, *P*2_1_/*c*	Mo *K*α radiation, λ = 0.71073 Å
*a* = 10.635 (5) Å	Cell parameters from 3686 reflections
*b* = 21.073 (10) Å	θ = 2.4–24.4°
*c* = 11.197 (5) Å	µ = 1.14 mm^−^^1^
β = 116.647 (5)°	*T* = 293 K
*V* = 2243.0 (18) Å^3^	Block, colorless
*Z* = 4	0.22 × 0.20 × 0.18 mm

### Data collection

**Table:**

Bruker SMART diffractometer	3992 independent reflections
Radiation source: fine-focus sealed tube	3424 reflections with *I* > 2σ(*I*)
graphite	*R*_int_ = 0.026
phi and ω scans	θ_max_ = 25.1°, θ_min_ = 1.9°
Absorption correction: multi-scan (*SADABS*; Sheldrick, 1996)	*h* = −10→12
*T*_min_ = 0.779, *T*_max_ = 0.815	*k* = −25→23
12067 measured reflections	*l* = −12→13

### Refinement

**Table:**

Refinement on *F*^2^	Primary atom site location: structure-invariant direct methods
Least-squares matrix: full	Secondary atom site location: difference Fourier map
*R*[*F*^2^ > 2σ(*F*^2^)] = 0.028	Hydrogen site location: inferred from neighbouring sites
*wR*(*F*^2^) = 0.077	H-atom parameters constrained
*S* = 1.03	*w* = 1/[σ^2^(*F*_o_^2^) + (0.0433*P*)^2^ + 0.4116*P*] where *P* = (*F*_o_^2^ + 2*F*_c_^2^)/3
3992 reflections	(Δ/σ)_max_ = 0.001
309 parameters	Δρ_max_ = 0.27 e Å^−^^3^
180 restraints	Δρ_min_ = −0.36 e Å^−^^3^

### Special details

**Table:**

Geometry. All e.s.d.'s (except the e.s.d. in the dihedral angle between two l.s. planes) are estimated using the full covariance matrix. The cell e.s.d.'s are taken into account individually in the estimation of e.s.d.'s in distances, angles and torsion angles; correlations between e.s.d.'s in cell parameters are only used when they are defined by crystal symmetry. An approximate (isotropic) treatment of cell e.s.d.'s is used for estimating e.s.d.'s involving l.s. planes.
Refinement. Refinement of *F*^2^ against ALL reflections. The weighted *R*-factor *wR* and goodness of fit *S* are based on *F*^2^, conventional *R*-factors *R* are based on *F*, with *F* set to zero for negative *F*^2^. The threshold expression of *F*^2^ > σ(*F*^2^) is used only for calculating *R*-factors(gt) *etc*. and is not relevant to the choice of reflections for refinement. *R*-factors based on *F*^2^ are statistically about twice as large as those based on *F*, and *R*- factors based on ALL data will be even larger.

### Fractional atomic coordinates and isotropic or equivalent isotropic displacement parameters (Å^2^)

**Table:**

	*x*	*y*	*z*	*U*_iso_*/*U*_eq_	
Zn1	0.73455 (2)	0.468990 (10)	0.79430 (2)	0.02980 (9)	
O2	0.91421 (15)	0.39030 (7)	0.67036 (15)	0.0479 (4)	
O5	0.95726 (15)	0.46026 (7)	0.87826 (14)	0.0394 (3)	
O1	0.70176 (15)	0.39837 (6)	0.66424 (14)	0.0382 (3)	
O3	0.73550 (16)	0.54552 (7)	0.69395 (15)	0.0462 (4)	
N1	0.51084 (17)	0.48044 (7)	0.73798 (16)	0.0332 (4)	
O4	0.84175 (16)	0.59595 (7)	0.88755 (14)	0.0452 (4)	
N2	0.71843 (17)	0.43150 (8)	0.96095 (16)	0.0329 (4)	
C2	0.7328 (2)	0.32287 (9)	0.52375 (18)	0.0347 (5)	
C9	0.8066 (2)	0.65093 (9)	0.68988 (18)	0.0313 (4)	
C1	0.7887 (2)	0.37425 (9)	0.62746 (18)	0.0328 (4)	
C8	0.79431 (19)	0.59369 (10)	0.7643 (2)	0.0337 (4)	
C19	0.4736 (2)	0.45195 (9)	0.82630 (19)	0.0305 (4)	
C22	0.6643 (2)	0.37283 (11)	1.1555 (2)	0.0473 (6)	
H22	0.6472	0.3532	1.2212	0.057*	
C20	0.5848 (2)	0.42643 (8)	0.94545 (19)	0.0298 (4)	
C10	0.7484 (2)	0.65085 (10)	0.5520 (2)	0.0466 (5)	
H10	0.7020	0.6150	0.5043	0.056*	
C17	0.2293 (3)	0.47341 (11)	0.6873 (2)	0.0519 (6)	
H17	0.1349	0.4707	0.6686	0.062*	
C18	0.3338 (2)	0.44721 (10)	0.8055 (2)	0.0382 (5)	
C21	0.5522 (2)	0.39749 (9)	1.0407 (2)	0.0363 (5)	
C24	0.8211 (2)	0.40768 (12)	1.0706 (2)	0.0462 (5)	
H24	0.9129	0.4109	1.0818	0.055*	
C7	0.5945 (2)	0.30438 (11)	0.4714 (2)	0.0503 (6)	
H7	0.5344	0.3244	0.4994	0.060*	
C3	0.8209 (2)	0.29284 (11)	0.4798 (2)	0.0450 (5)	
H3	0.9145	0.3053	0.5130	0.054*	
C26	0.4092 (2)	0.39395 (11)	1.0174 (2)	0.0457 (5)	
H26	0.3878	0.3751	1.0811	0.055*	
C25	0.3045 (2)	0.41735 (11)	0.9048 (2)	0.0477 (6)	
H25	0.2120	0.4140	0.8915	0.057*	
C15	0.4098 (2)	0.50578 (11)	0.6288 (2)	0.0421 (5)	
H15	0.4345	0.5263	0.5689	0.050*	
C14	0.8760 (2)	0.70456 (10)	0.7581 (2)	0.0431 (5)	
H14	0.9174	0.7049	0.8510	0.052*	
C23	0.7978 (2)	0.37786 (13)	1.1703 (2)	0.0546 (6)	
H23	0.8729	0.3616	1.2459	0.066*	
C16	0.2681 (2)	0.50307 (12)	0.5999 (2)	0.0518 (6)	
H16	0.2003	0.5212	0.5219	0.062*	
C13	0.8849 (3)	0.75778 (11)	0.6907 (2)	0.0535 (6)	
H13	0.9309	0.7938	0.7380	0.064*	
C4	0.7690 (3)	0.24424 (12)	0.3863 (2)	0.0549 (6)	
H4	0.8285	0.2239	0.3580	0.066*	
C12	0.8259 (3)	0.75749 (11)	0.5539 (3)	0.0552 (6)	
H12	0.8310	0.7934	0.5081	0.066*	
C11	0.7593 (3)	0.70405 (12)	0.4849 (2)	0.0607 (7)	
H11	0.7210	0.7036	0.3922	0.073*	
C5	0.6316 (3)	0.22613 (12)	0.3356 (2)	0.0569 (7)	
H5	0.5976	0.1936	0.2730	0.068*	
C6	0.5433 (3)	0.25633 (13)	0.3777 (2)	0.0621 (7)	
H6	0.4493	0.2443	0.3429	0.075*	
H5B	1.0206	0.4450	0.9561	0.078 (9)*	
H5A	0.9702	0.4340	0.8221	0.087 (10)*	

### Atomic displacement parameters (Å^2^)

**Table:**

	*U*^11^	*U*^22^	*U*^33^	*U*^12^	*U*^13^	*U*^23^
Zn1	0.03210 (15)	0.03006 (14)	0.03060 (14)	−0.00163 (9)	0.01704 (11)	0.00108 (9)
O2	0.0355 (9)	0.0604 (10)	0.0473 (9)	−0.0013 (7)	0.0181 (7)	−0.0157 (7)
O5	0.0324 (8)	0.0498 (9)	0.0337 (8)	0.0002 (6)	0.0128 (7)	−0.0041 (6)
O1	0.0371 (8)	0.0398 (8)	0.0400 (8)	−0.0024 (6)	0.0194 (7)	−0.0094 (6)
O3	0.0459 (9)	0.0379 (9)	0.0469 (9)	−0.0105 (7)	0.0137 (7)	0.0091 (7)
N1	0.0360 (9)	0.0313 (9)	0.0347 (9)	0.0013 (7)	0.0180 (8)	0.0012 (7)
O4	0.0547 (10)	0.0523 (9)	0.0371 (8)	0.0132 (7)	0.0282 (8)	0.0134 (7)
N2	0.0316 (9)	0.0352 (9)	0.0341 (9)	0.0006 (7)	0.0167 (7)	0.0031 (7)
C2	0.0432 (12)	0.0321 (11)	0.0279 (10)	0.0038 (9)	0.0150 (9)	0.0017 (8)
C9	0.0289 (10)	0.0326 (11)	0.0340 (10)	0.0012 (8)	0.0155 (8)	0.0048 (8)
C1	0.0360 (12)	0.0322 (11)	0.0291 (10)	0.0065 (9)	0.0135 (9)	0.0043 (8)
C8	0.0260 (10)	0.0374 (12)	0.0406 (12)	0.0052 (8)	0.0175 (9)	0.0083 (9)
C19	0.0337 (11)	0.0265 (10)	0.0358 (10)	0.0005 (8)	0.0196 (9)	−0.0026 (8)
C22	0.0525 (15)	0.0537 (14)	0.0443 (13)	0.0055 (11)	0.0294 (11)	0.0144 (10)
C20	0.0327 (11)	0.0260 (10)	0.0360 (10)	−0.0009 (8)	0.0200 (9)	−0.0026 (8)
C10	0.0559 (15)	0.0390 (13)	0.0373 (12)	−0.0062 (10)	0.0142 (11)	0.0027 (9)
C17	0.0344 (12)	0.0613 (16)	0.0593 (15)	0.0057 (11)	0.0205 (11)	0.0021 (12)
C18	0.0326 (11)	0.0383 (12)	0.0455 (12)	0.0014 (9)	0.0192 (10)	−0.0006 (9)
C21	0.0419 (12)	0.0343 (11)	0.0400 (11)	−0.0008 (9)	0.0249 (10)	0.0016 (9)
C24	0.0335 (12)	0.0637 (15)	0.0434 (13)	0.0043 (10)	0.0190 (10)	0.0112 (11)
C7	0.0502 (14)	0.0567 (15)	0.0475 (13)	−0.0043 (11)	0.0252 (11)	−0.0143 (11)
C3	0.0478 (13)	0.0474 (13)	0.0354 (11)	0.0109 (10)	0.0148 (10)	−0.0010 (10)
C26	0.0479 (13)	0.0478 (14)	0.0564 (14)	−0.0003 (10)	0.0368 (12)	0.0079 (10)
C25	0.0363 (12)	0.0556 (15)	0.0616 (15)	−0.0015 (10)	0.0311 (12)	0.0032 (11)
C15	0.0454 (13)	0.0452 (13)	0.0362 (11)	0.0059 (10)	0.0187 (10)	0.0059 (9)
C14	0.0528 (14)	0.0405 (13)	0.0375 (11)	−0.0040 (10)	0.0215 (10)	−0.0013 (9)
C23	0.0466 (14)	0.0753 (18)	0.0417 (13)	0.0133 (12)	0.0197 (11)	0.0231 (12)
C16	0.0417 (13)	0.0607 (15)	0.0447 (13)	0.0103 (11)	0.0121 (11)	0.0082 (11)
C13	0.0660 (17)	0.0355 (13)	0.0641 (16)	−0.0105 (11)	0.0338 (14)	−0.0038 (11)
C4	0.0730 (18)	0.0499 (15)	0.0419 (13)	0.0205 (13)	0.0258 (13)	−0.0031 (10)
C12	0.0735 (18)	0.0376 (13)	0.0659 (16)	0.0018 (11)	0.0415 (14)	0.0155 (11)
C11	0.0805 (19)	0.0593 (17)	0.0395 (13)	0.0022 (14)	0.0246 (13)	0.0156 (11)
C5	0.083 (2)	0.0422 (14)	0.0417 (13)	−0.0056 (13)	0.0243 (13)	−0.0106 (10)
C6	0.0618 (17)	0.0668 (18)	0.0562 (16)	−0.0205 (13)	0.0249 (13)	−0.0211 (13)

### Geometric parameters (Å, °)

**Table:**

Zn1—O3	1.9681 (16)	C17—C18	1.405 (3)
Zn1—O1	2.0003 (15)	C17—H17	0.9300
Zn1—N2	2.1030 (17)	C18—C25	1.428 (3)
Zn1—O5	2.1285 (17)	C21—C26	1.425 (3)
Zn1—N1	2.184 (2)	C24—C23	1.396 (3)
O2—C1	1.246 (2)	C24—H24	0.9300
O5—H5B	0.8887	C7—C6	1.383 (3)
O5—H5A	0.8928	C7—H7	0.9300
O1—C1	1.274 (2)	C3—C4	1.390 (3)
O3—C8	1.265 (3)	C3—H3	0.9300
N1—C15	1.325 (3)	C26—C25	1.346 (3)
N1—C19	1.359 (2)	C26—H26	0.9300
O4—C8	1.239 (2)	C25—H25	0.9300
N2—C24	1.322 (3)	C15—C16	1.393 (3)
N2—C20	1.357 (2)	C15—H15	0.9300
C2—C7	1.373 (3)	C14—C13	1.378 (3)
C2—C3	1.391 (3)	C14—H14	0.9300
C2—C1	1.502 (3)	C23—H23	0.9300
C9—C14	1.378 (3)	C16—H16	0.9300
C9—C10	1.382 (3)	C13—C12	1.370 (3)
C9—C8	1.505 (3)	C13—H13	0.9300
C19—C18	1.401 (3)	C4—C5	1.363 (4)
C19—C20	1.433 (3)	C4—H4	0.9300
C22—C23	1.358 (3)	C12—C11	1.370 (4)
C22—C21	1.404 (3)	C12—H12	0.9300
C22—H22	0.9300	C11—H11	0.9300
C20—C21	1.400 (3)	C5—C6	1.381 (3)
C10—C11	1.383 (3)	C5—H5	0.9300
C10—H10	0.9300	C6—H6	0.9300
C17—C16	1.372 (3)		
			
O3—Zn1—O1	103.76 (7)	C17—C18—C25	123.3 (2)
O3—Zn1—N2	146.90 (7)	C20—C21—C22	117.41 (19)
O1—Zn1—N2	108.03 (7)	C20—C21—C26	119.46 (19)
O3—Zn1—O5	91.91 (6)	C22—C21—C26	123.13 (18)
O1—Zn1—O5	93.06 (6)	N2—C24—C23	123.0 (2)
N2—Zn1—O5	95.34 (6)	N2—C24—H24	118.5
O3—Zn1—N1	91.63 (6)	C23—C24—H24	118.5
O1—Zn1—N1	93.46 (6)	C2—C7—C6	120.9 (2)
N2—Zn1—N1	77.64 (6)	C2—C7—H7	119.6
O5—Zn1—N1	171.63 (6)	C6—C7—H7	119.6
Zn1—O5—H5B	131.9	C4—C3—C2	119.9 (2)
Zn1—O5—H5A	103.6	C4—C3—H3	120.0
H5B—O5—H5A	100.3	C2—C3—H3	120.0
C1—O1—Zn1	128.11 (13)	C25—C26—C21	121.24 (19)
C8—O3—Zn1	115.44 (13)	C25—C26—H26	119.4
C15—N1—C19	117.96 (18)	C21—C26—H26	119.4
C15—N1—Zn1	129.69 (14)	C26—C25—C18	120.9 (2)
C19—N1—Zn1	112.11 (13)	C26—C25—H25	119.6
C24—N2—C20	118.04 (17)	C18—C25—H25	119.6
C24—N2—Zn1	127.20 (14)	N1—C15—C16	122.9 (2)
C20—N2—Zn1	114.44 (12)	N1—C15—H15	118.6
C7—C2—C3	118.9 (2)	C16—C15—H15	118.6
C7—C2—C1	120.76 (18)	C9—C14—C13	121.0 (2)
C3—C2—C1	120.37 (19)	C9—C14—H14	119.5
C14—C9—C10	118.68 (19)	C13—C14—H14	119.5
C14—C9—C8	120.55 (18)	C22—C23—C24	119.2 (2)
C10—C9—C8	120.76 (18)	C22—C23—H23	120.4
O2—C1—O1	125.05 (18)	C24—C23—H23	120.4
O2—C1—C2	118.57 (17)	C17—C16—C15	119.6 (2)
O1—C1—C2	116.39 (18)	C17—C16—H16	120.2
O4—C8—O3	124.40 (19)	C15—C16—H16	120.2
O4—C8—C9	119.53 (19)	C12—C13—C14	119.9 (2)
O3—C8—C9	116.07 (17)	C12—C13—H13	120.0
N1—C19—C18	122.98 (18)	C14—C13—H13	120.0
N1—C19—C20	117.09 (17)	C5—C4—C3	120.6 (2)
C18—C19—C20	119.93 (17)	C5—C4—H4	119.7
C23—C22—C21	119.64 (19)	C3—C4—H4	119.7
C23—C22—H22	120.2	C11—C12—C13	119.7 (2)
C21—C22—H22	120.2	C11—C12—H12	120.1
N2—C20—C21	122.68 (18)	C13—C12—H12	120.1
N2—C20—C19	117.99 (16)	C12—C11—C10	120.5 (2)
C21—C20—C19	119.32 (17)	C12—C11—H11	119.7
C9—C10—C11	120.1 (2)	C10—C11—H11	119.7
C9—C10—H10	120.0	C4—C5—C6	119.7 (2)
C11—C10—H10	120.0	C4—C5—H5	120.2
C16—C17—C18	119.1 (2)	C6—C5—H5	120.2
C16—C17—H17	120.4	C5—C6—C7	120.0 (2)
C18—C17—H17	120.4	C5—C6—H6	120.0
C19—C18—C17	117.46 (19)	C7—C6—H6	120.0
C19—C18—C25	119.19 (19)		

### Hydrogen-bond geometry (Å, °)

**Table:**

*D*—H···*A*	*D*—H	H···*A*	*D*···*A*	*D*—H···*A*
O5—H5A···O2	0.89	1.78	2.616 (2)	154.
O5—H5B···O4^i^	0.89	1.91	2.797 (2)	174.

Symmetry codes: (i) −*x*+2, −*y*+1, −*z*+2.
